# Object Clusters or Spectral Energy? Assessing the Relative Contributions of Image Phase and Amplitude Spectra to Trypophobia

**DOI:** 10.3389/fpsyg.2020.01847

**Published:** 2020-07-24

**Authors:** R. Nathan Pipitone, Christopher DiMattina

**Affiliations:** Department of Psychology, Florida Gulf Coast University, Fort Myers, FL, United States

**Keywords:** trypophobia, visual discomfort, Fourier analysis, phase spectrum, amplitude spectrum

## Abstract

Trypophobia refers to the visual discomfort experienced by some people when viewing clustered patterns (e.g., clusters of holes). Trypophobic images deviate from the 1/*f* amplitude spectra typically characterizing natural images by containing excess energy at mid-range spatial frequencies. While recent work provides partial support for the idea of excess mid-range spatial frequency energy causing visual discomfort when viewing trypophobic images, a full factorial manipulation of image phase and amplitude spectra has yet to be conducted in order to determine whether the phase spectrum (sinusoidal waveform patterns that comprise image details like edge and texture elements) also plays a role in trypophobic discomfort. Here, we independently manipulated the phase and amplitude spectra of 31 Trypophobic images using a standard Fast Fourier Transform (FFT). Participants rated the four different versions of each image for levels of visual comfort, and completed the Trypophobia Questionnaire (TQ). Images having the original phase spectra intact (with either original or 1/*f* amplitude) explained the most variance in comfort ratings and were rated lowest in comfort. However, images with the original amplitude spectra but scrambled phase spectra were rated higher in comfort, with a smaller amount of variance in comfort attributed to the amplitude spectrum. Participant TQ scores correlated with comfort ratings only for images having the original phase spectra intact. There was no correlation between TQ scores and comfort levels when participants viewed the original amplitude / phase-scrambled images. Taken together, the present findings show that the phase spectrum of trypophobic images, which determines the pattern of small clusters of objects, plays a much larger role than the amplitude spectrum in determining visual discomfort.

## Introduction

**FIGURE 1:**
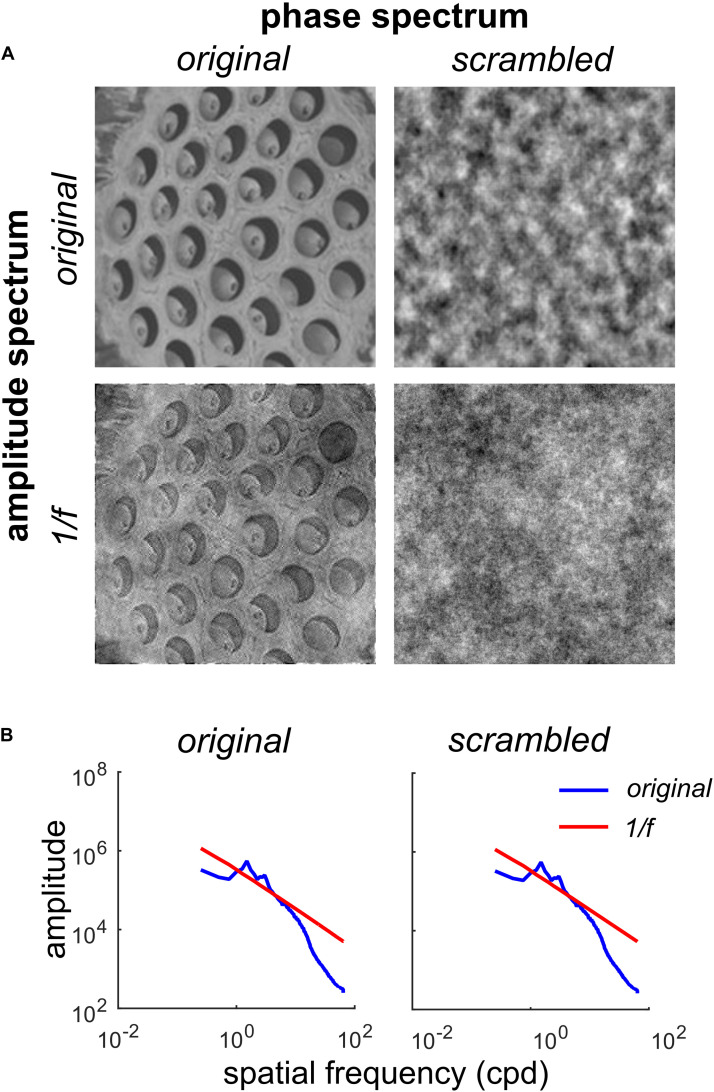
Factorial manipulation of amplitude and phase spectra for a representative trypophobic image. **(A)** Top left: The original image. Top right: Original amplitude spectrum, scrambled phase spectrum. Note the absence of fine edge structure needed to define holes. Bottom left: Original phase spectrum, 1/*f* amplitude spectrum. Note the holes are still clearly visible, but have lower contrast. Bottom right: 1/*f* noise. **(B)** Left: Rotational average of the amplitude spectra of the original image (blue) and original phase / 1/*f* amplitude image (red line) shown in **(A)**. Right: Rotational average of the amplitude spectra of the original amplitude/scrambled phase (blue) and 1/*f* noise (red) images shown in **(A)**.

Trypophobia is a recently documented perceptual phenomenon characterized by extreme negative reactions when viewing repetitive clusters of objects, usually holes or bumps (Cole and Wilkins, 2013; Le et al., 2015). An example of a trypophobic image is shown in Figure 1A (top left). A small but sizable proportion of individuals qualify as being trypophobic (roughly 15–17%, Cole and Wilkins, 2013; Pipitone et al., 2017), and furthermore, many non-trypophobic individuals (Cole and Wilkins, 2013; Kupfer and Le, 2017; Pipitone et al., 2017) including children (Can et al., 2017) report experiencing some level of discomfort when viewing trypophobic images. Since its initial description in the scientific literature (Cole and Wilkins, 2013) there has been strong interest in trypophobia in both the scientific community and the popular media, with the Washington Post recently reporting that the cluster of camera lenses on Apple’s new iPhone 11 may be triggering trypophobia (Shepherd, 2019).

Several theoretical frameworks have been proposed to explain trypophobia (Cole and Wilkins, 2013; Kupfer and Le, 2017; Sasaki et al., 2017) and suggest that the discomfort elicited by trypophobic stimuli is an evolved response to help organisms detect and avoid harmful stimuli. One proposal is that the characteristic trypophobic patterns contain excessive energy at mid-range spatial frequencies, as defined in previous work (Sasaki et al., 2017: 2–9 cycles per degree of visual angle (cpd); Fernandez and Wilkins, 2008: 3 cpd ±2 octaves). This same spectral energy profile can also be observed in the patterning of many venomous and/or predatory animals like snakes and spiders, provided that these stimuli are viewed at the appropriate distance (American Psychiatric Association, 2013). In contrast, an alternative theory proposes that the aversion to clusters of roughly circular shapes represents a response that helps organisms avoid parasitism and infectious disease (Kupfer and Le, 2017). Some of the previous work suggests that the negative reactions from viewing trypophobic images stems from patterns that contain high contrast energy at mid-range spatial frequencies. However, several recent findings raise the possibility of other interpretations. Using image filtering techniques, Sasaki et al. (2017) showed that low to mid-range spatial frequencies in trypophobic images invoked the most discomfort, comparable to the original images, but specifically filtering mid-range spatial frequencies did not reduce discomfort. What is more, this study did not account for the image phase spectrum. Le et al. (2015) filtered trypophobic images to have a 1/*f* natural image spectra, thus removing the excess mid-range energy, and found that those images continued to invoke discomfort. Using a continuous flash suppression technique to measure access to early visual awareness, Shirai and Ogawa (2019) recently showed that trypophobic images enter visual awareness earlier than fearful or neutral images, but a second experiment in which these images were phase-scrambled (but the original amplitude spectrum was left intact) mitigated the early awareness effect.

Another issue with the spectral energy hypothesis of trypophobia (Cole and Wilkins, 2013) is that image spatial frequencies shift as image size or viewing distance changes. For example, if one doubles viewing distance, an image subtends roughly half its original angle on the retina, so that its spatial frequency content approximately doubles. Having protective reactions (i.e., discomfort) elicited only by specific spatial frequencies of potentially harmful stimuli (whether disease, or predatory) might limit their protective ability. These recent findings coupled with the viewing distance issue suggest that the energy spectra of trypophobic images may not be solely responsible for evoking trypophobic discomfort.

### Present Study

This study seeks to better understand the relationship between the spectral components of trypophobic images and their relation to levels of viewing comfort. Using Fourier analysis, the phase and amplitude spectra of trypophobic images were independently manipulated in a factorial design to assess the unique aspects of each on levels of comfort. Based on previous work that has shown discomfort to trypophobic images even with mid-range frequencies experimentally removed, coupled with the fact that viewing distance plays an important role in frequency parameters, we hypothesized that the phase spectrum of trypophobic images (the sinusoidal waveform patterns that comprise fine details like edge and texture elements) also play a role in eliciting trypophobic reactions. Following Sasaki et al. (2017) we then categorized participants into high and low trypophobia groups [using the Trypophobia Questionnaire (TQ)] to assessed whether TQ levels impact viewing comfort to the different image categories. Finally, based on previous work (Le et al., 2015; Pipitone et al., 2017) we assessed how viewing comfort changes as participant’s level of trypophobia changes across the four image manipulations. In addition to these main issues, we also tested whether or not subjective image ratings were robust to monitor properties and were consistent across different instances of our random phase-scrambling procedure.

## Materials and Methods

### Images and Image Manipulation

This study was approved by the Florida Gulf Coast University Institutional Review Board (IRB). All methods were carried out in accordance with the IRB’s guidelines and regulations, and informed consent was obtained from all participants. Thirty-one trypophobic images (T1-T31) were used in this study. Some images were obtained from websites devoted to trypophobia (e.g., https://trypophobia.com/), while other images were provided by Arnold Wilkins and were used in Cole and Wilkins (2013), Pipitone et al. (2017). All images were cropped to minimize non-trypophobic background imagery, resized to 512 × 512 pixels, converted to grayscale, scaled to 25% RMS contrast, and saved as 8-bit BMP files.

In order to independently manipulate the phase and amplitude spectra of the images, we employed the standard 2-D Fast Fourier Transform (FFT) implemented in MATLAB^®^ (R2015a) as fft2.m. Fourier image analysis makes use of the mathematical fact that every image can be exactly reconstructed using a weighted linear sum of sinusoidal plane waves having varying orientations, spatial frequencies and phases. The set of weights applied to each plane wave for a given image are collectively known as the *amplitude spectrum*, and the phases of each plane wave are known as the *phase spectrum*. Therefore, we can equivalently characterize an image in either the space domain (pixel values) or the frequency domain (amplitude and phase spectra), using the FFT and the inverse FFT to map between the two (Gonzalez and Woods, 2017). The amplitude spectra defines which spatial frequencies are present in an image, and the phase spectrum determines where the waves interfere constructively and destructively to create localized features like edges or texture elements (Oppenheim and Lim, 1981; Tadmor and Tolhurst, 1993; Wichmann et al., 2006). Therefore, by manipulating the phase spectrum while leaving the amplitude spectrum intact, we can create an image with the same spatial frequency content as a trypophobic image, but with no localized visual patterns like holes or bumps. Conversely, by manipulating the amplitude spectrum while leaving the phase spectrum intact, we can create images with a 1/*f* amplitude typical of most natural images (Field, 1987; Ruderman and Bialek, 1994) but having the same clustered object pattern as trypophobic images. Hence, we can dissociate the clustered pattern from the amplitude spectrum to evaluate their relative contributions to levels of viewing comfort.

Figure 1A illustrates our manipulations of one of the trypophobic images (T4). The original image can be found in the top left corner of Figure 1A.

By randomizing the phase spectrum (uniform distribution, −180 to +180°), while preserving the original amplitude spectrum, we produce the phase-scrambled image (Figure 1A, top right). Note that while this image has the same spatial auto-correlation structure as the original, it does not have the fine edge structure which defines the clustered pattern of holes in the original. As we see in Figure 1B, this phase-scrambled image has an identical amplitude spectrum to the original image (blue curves in both panels), with both images containing an excess of mid-range spatial frequency energy relative to 1/*f* (Figure 1B, red curves). Conversely, we can take the original image and set its amplitude spectrum to the 1/*f* spectrum typical of natural images. This produces the image in the lower left of Figure 1A, which contains the fine structure of the original, including the holes, but has a somewhat “washed out” appearance. Finally, as a control stimulus, we also present 1/*f* noise (Figure 1A, bottom right) which has a naturalistic amplitude spectrum and also a randomized phase spectrum. Collectively, this set of images defines a 2 × 2 factorial design where the first factor is the amplitude spectrum (original or 1/*f*) and the second factor is the phase spectrum (original or scrambled).

In order to verify that the 31 Trypophobic images in our set contain excess spectral energy (relative to 1/*f*) in mid-range spatial frequencies, for each image we computed the proportion of total stimulus energy in each of the two mid-range spatial frequency bands defined previously (Sasaki et al., 2017: 2–9 cpd; Fernandez and Wilkins, 2008: 3 cpd ±2 octaves, or 0.75–12 cpd). We then computed the proportion of stimulus energy in these same frequency ranges for 1/*f* images, and took a ratio ρ of the proportions computed for our trypophobic images to those computed for the 1/*f* images. A ratio ρ > 1 represents excessive mid-range spatial frequency energy with respect to 1/*f*.

**FIGURE 2:**
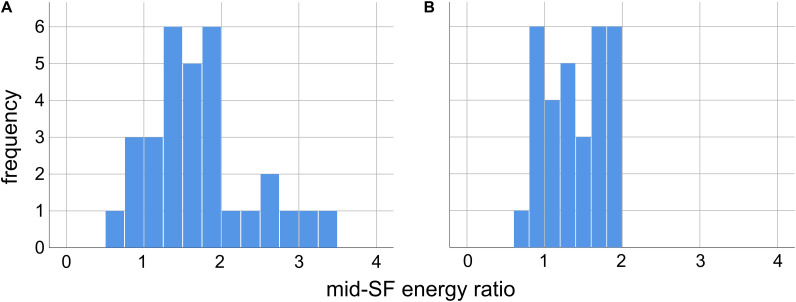
Ratio of the mid-range spatial frequency (SF) energy in our 31 images relative to energy in the same SF range in images with 1/*f* spectra, for two different definitions of mid-range SF. A ratio greater than unity indicates excess energy in this range relative to 1/*f*. **(A)** Mid-range SF defined as 2–9 cycles per degree of visual angle, as in Sasaki et al. (2017). **(B)** Mid-range SF defined as 3 cpd, ±2 octaves (0.75–12 cpd), as in Fernandez and Wilkins (2008).

As we can see from the histograms in Figure 2, for both definitions of mid-range spatial frequencies, the overwhelming majority of the images in our set have a greater proportion of energy in these ranges than 1/*f* images. For the 2–9 cpd definition (Sasaki et al., 2017) we see in Figure 2A that 27/31 images have a ρ > 1, with median ρ = 1.67 (*Q*_1_ = 1.27, *Q*_3_ = 1.92) significantly greater than unity (*p* < 0.001, Wilcoxon sign-rank test, *N* = 31). Likewise, for the 3 cpd ±2 octaves (or 0.75–12 cpd) proposed by Fernandez and Wilkins (2008) we see in Figure 2B that 24/31 images have ρ > 1, with median ρ = 1.40 (*Q*_1_ = 1.01, *Q*_3_ = 1.75) significantly greater than unity (*p* < 0.001, Wilcoxon sign-rank test, *N* = 31). Across the set of images we find a significant positive correlation between the values of ρ obtained from the two definitions of mid-range spatial frequencies [*r*(31) = 0.67, *p* < 0.001], with the value measured using one definition accounting for about 45% of the total variance in the value measured with the other definition (*r*^2^ = 0.449).

### Participants

One hundred and forty seven undergraduate students (34 male, 113 female) ranging from 18 to 30 years in age (*M* = 19.65, *SD* = 2.15, with 7 not reporting age) were recruited from the institution’s General Psychology research pool (Sona-Systems^®^)^1^ for class credit or from other classrooms for extra credit. All participants reported having normal or corrected-to-normal vision. *A priori* power calculations were conducted using G^∗^Power 3.1 beforehand to estimate sample sizes needed to achieve 80% power for the most stringent tests. In our study, the tests that had the smallest *a-priori* sample sizes were the mixed model ANOVAs with TRY condition as a between-subjects factor and also the correlations between comfort level and TQ scores among high and low TRY individuals. For the mixed model ANOVAs, using a medium effect size and an alpha of 0.05, the calculated sample size needed to achieve 80% power was 82 (we had 98). For the tests using Pearson correlation coefficients, we used a large effect size as the determinant based on previous literature (Le et al., 2015; Pipitone et al., 2017). The most stringent tests were going to be the correlations between comfort levels and TQ scores among high and low TRY participants. In G^∗^power using a large effect size, two-tailed test, and an alpha of 0.05, the sample size needed to achieve 80% power was 29. Our smallest test has 46 participants [low trypophobia condition (LTRY)] hence we had sufficient power here as well.

### Procedure

Participants used the online survey Checkbox^®2^ to answer several demographic questions (e.g., history of psychological disorders, any medication, normal vision) and the GAD-7 for generalized anxiety levels (Spitzer et al., 2006). Then, participants viewed two alternating trypophobic images on another computer monitor (lotus seedpod and honeycomb) and completed the TQ (Le et al., 2015) which has 17 items assessing various emotional reactions to the images (e.g., feel nervous, feel sick or nauseous, feel skin crawl) on a five-point Likert scale (ranging from not at all to extremely). Responses to both the GAD-7 (range 0–21, *M* = 5.86, *SD* = 4.61) and TQ (range 17–85, *M* = 21.12, *SD* = 6.74) were summed to create aggregate levels of anxiety and trypophobia, respectively. Participants then viewed the original and three different manipulated versions of 31 trypophobic images on one computer (four stimuli for each original image: see Figure 1) and were given 7 s for each stimulus to rate their level of comfort on a scale of −5 (extremely uncomfortable) to 5 (extremely comfortable) using Checkbox^®^ survey software running on another computer. Since different instantiations of the stimuli having randomized phase spectra yield non-identical images, the first 20 participants viewed two different versions of the phase-scrambled and 1/*f* noise images (Figure 1A, right column), for a total of six stimuli per original image.

Previous work on trypophobia has used highly variable visual displays and has presented images at a variety of spatial scales (Cole and Wilkins, 2013; Le et al., 2015; Sasaki et al., 2017). Given the average size and periodicity of the clusters in our images, and the size of the images on the monitors, viewing distance from the monitors was chosen so that the images subtended four degrees of visual angle (dva). This focused the excess spectral energy for each image in roughly in the same range (∼3 cpd) described previously as inducing discomfort (Fernandez and Wilkins, 2008; Cole and Wilkins, 2013; Sasaki et al., 2017). In addition, since previous work has not systematically investigated whether monitor gamma calibration affects trypophobic responses, we ran our tests on both an uncalibrated LCD monitor (91 participants) and two different gamma-corrected CRT monitors (56 participants). The uncalibrated LCD monitor was a Dell P2213 (22″, 1680 × 1050), viewed at a distance of 158 cm. Images on this set-up were presented as a slide-show using PowerPoint^®^. Two different gamma-corrected (gamma = 1.0) monitors driven by a Bits# stimulus processor (Cambridge Research) were employed. The first gamma-corrected setup was a ViewSonic^®^ Optiquest Q71 (17″, 1024 × 768, 75 Hz) with midpoint luminance of 52.2 cd/m^2^. Images were scaled to 256 × 256 and viewed at a distance of 108 cm. The second gamma-corrected setup was a SONY FD-500 Trinitron (21″, 1024 × 768, 75 Hz) having midpoint luminance of 35.6 cd/m^2^. Images were scaled to 256 × 256 and viewed at distance of 143 cm. Stimulus presentation for both of these calibrated setups was controlled by a custom MATLAB^®^ script employing PsychToolbox-3 routines (Kleiner et al., 2007).

## Results

Data were analyzed using the GLM procedure in SPSS^®^ version 24.0 (IBM). Twenty seven participants (18.4%) reported suffering from a clinical psychological disorder (e.g., depression, anxiety, comorbid depression and anxiety). Of these participants, 13 were currently using medication. Exclusion of these participants did not change any of the results, thus they were included in all subsequent analyses. Outliers were established using Z cutoff values outside of 3.33 standard deviation units (Tabachnick and Fidell, 2007).

### Effects of Phase and Amplitude on Comfort Levels

**FIGURE 3:**
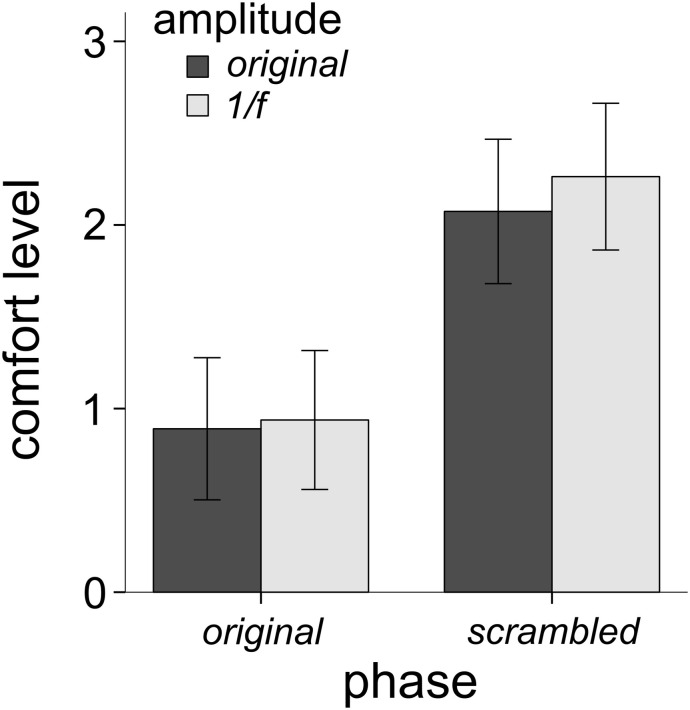
Mean comfort levels across all 31 trypophobic images (and their three manipulated versions) for all 146 participants (error bars represent 95% confidence intervals). The main effect of phase explained the most variance in comfort (24.9% variance explained), followed by the main effect of amplitude (9% variance explained), followed by the interaction of phase and amplitude (6.1% variance explained).

Figure 3 shows the average comfort ratings for each of the four image conditions (Figure 1A) for all participants. A 2 (phase condition: original vs. scrambled) × 2 (amplitude condition: original vs. 1/*f*) repeated measures factorial ANOVA was performed to examine the unique impact of phase and amplitude on levels of comfort. There was a significant main effect of the phase spectrum on comfort, *F*(1,146) = 48.39, *p* < 0.001, η^2^ = 0.249. Participants rated the original-phase images lower in comfort than the scrambled-phase images. There was also a significant main effect of the amplitude spectrum on comfort, *F*(1,146) = 14.51, *p* < 0.001, η^2^ = 0.09. As shown in Figure 3, average comfort ratings were lower for the original-amplitude images compared to the 1/*f* amplitude images. There was also a significant interaction of the phase and amplitude spectra on comfort, *F*(1,146) = 9.54, *p* = 0.002, η^2^ = 0.061. Participants gave slightly lower comfort ratings to the scrambled phase / original amplitude images than the scrambled phase / 1/*f* images, but there was little difference between the ratings given to the original images and the original phase / 1/*f* amplitude images.

One possible consequence of scrambling the phase property of images is that it leads them to be more comfortable to view in general. In order to test this, we ran a separate control study on 45 participants (9 males, 36 females, ages *M* = 20.3, *SD* = 2.41). We used 15 non-trypophobic control images of holes (e.g., pictures of a circular window, golf hole, cannon barrel) from Cole and Wilkins (2013) along with their phase-scrambled, original amplitude counterparts. Results showed that scrambling the phase property of control hole images actually decreases comfort levels (*M* = 1.49, *SD* = 2.44) compared to the original control images of holes (*M* = 2.34, *SD* = 1.92), *t*(44) = 3.56, *p* = 0.001, η^2^ = 0.23. There was no effect of participant level of trypophobia based on high and low TQ scores and image condition, *F*(1,43) = 0.26, *p* = 0.613, η^2^ = 0.01. These results show that phase scrambling images does not increase their comfort level, in fact it has the opposite effect. Therefore, it is unlikely that the increased comfort level attributed to phase-scrambled trypophobic images was due to scrambling of their phase properties.

### Controls for Noise Instance and Gamma Correction

In order to assess whether our effects were consistent across different instantiations of the random phase spectra used to generate the scrambled phase and 1/*f* noise conditions, the first 20 participants viewed two different scrambled phase (Figure 1A, top right) and 1/*f* amplitude (Figure 1A, bottom right) images per original trypophobic image (for a total of six stimuli per original image rather than four). Using the images as the level of analysis (each image was rated by 20 participants), two paired-sample *t*-tests showed that levels of comfort were not significantly different across all of the first vs. second scrambled phase manipulated images, *t*(30) = −0.751, *p* = 0.459, or the first vs. second 1/*f* amplitude images, *t*(30) = −0.289, *p* = 0.774. Using the raters as the level of analysis across all 62 image types, paired sample *t*-tests between all of the 31 first vs. second scrambled phase and 31 first vs. second 1/*f* amplitude images revealed only one significant difference in comfort (image T30 for first vs. second 1/*f* amplitude), but this is well within the range of the expected Type I error inflation from running 62 tests. We conclude that no manipulation effects were evident when constructing the scrambled phase and 1/*f* amplitude images, hence the full analysis only used the first generated scrambled phase and 1/*f* amplitude image.

We also assessed whether responses would change as a function of monitor gamma correction, an issue that has not been explored previously. Using a mixed-model factorial ANOVA with monitor type as a between-subjects factor, the main effect of monitor type alone did not influence levels of comfort, *F*(1,145) = 2.193, *p* = 0.141, η^2^ = 0.015. The three-way interaction of phase, amplitude, and monitor type on levels of comfort was also not significant, nor were the two-way interactions of phase and monitor type or amplitude and monitor type, all *F*’s < 1.928, *p* > 0.167, η^2^ < 0.013). We conclude that our observed effects of amplitude and phase spectra on comfort levels are robust to the gamma non-linearity present in standard commercial displays (Lu and Dosher, 2013). Therefore, for all other analyses reported here we pooled the data across both gamma calibrated and un-calibrated setups.

### TQ Scores, Phase, and Amplitude on Comfort Levels

**FIGURE 4:**
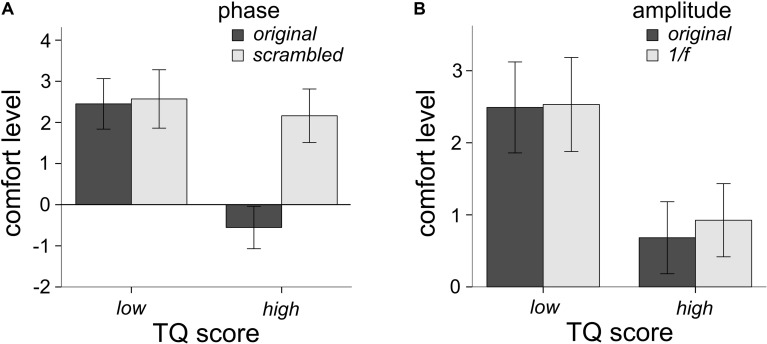
Interactions between TQ scores and the phase spectra **(A)** and TQ scores and the amplitude spectra **(B)** on levels of comfort. The TQ by phase interaction was the largest effect, explaining 36.1% of the variance in comfort levels. The TQ by amplitude interaction explained 8.1% of the variance in comfort levels. Participants who scored high on the TQ rated images with the phase spectra intact much lower in comfort **(A)**, compared to other conditions. Error bars represent 95% confidence intervals.

Participants filled out the TQ, in order to assess whether levels of trypophobia would affect ratings of comfort as a function of image phase and amplitude spectra. Eleven participants had scores higher than 31 (7.4%) and would be considered trypophobic (Le et al., 2015) which is a smaller proportion than what others have reported in their samples (Cole and Wilkins, 2013; Pipitone et al., 2017). Following Sasaki et al. (2017) we ran analyses on participants who scored in the top and bottom third (33%) of the TQ. As a result, 49 participants were in the high trypophobia condition (HTRY) and 49 were in the LTRY. Three participants had outlier raw TQ scores (Z > 3.33), however, their comfort ratings were not outliers, hence their data is retained in this analysis (see later analyses for their exclusion). Using a mixed-model factorial ANOVA with TRY condition (LTRY and HTRY) as the between-subjects factor, the main effect of TRY condition did influence levels of comfort, *F*(1,96) = 17.63, *p* < 0.001, η^2^ = 0.155. HTRY participants had lower levels of comfort than LTRY participants, which is to be expected. The three-way interaction of phase, amplitude and TRY condition was not significant, *F*(1,96) = 2.89, *p* = 0.092, η^2^ = 0.029. However, the two-way interaction of phase and TRY condition on levels of comfort was significant *F*(1,96) = 54.17, *p* < 0.001, η^2^ = 0.361. As seen in Figure 4A, LTRY participants had similar comfort scores when viewing original or scrambled phase images, collapsed across the amplitude condition, but HTRY participants had lower comfort levels when viewing the original phase images compared to the scrambled phase images, collapsed across amplitude condition. The two-way interaction of amplitude and TRY condition on levels of discomfort was also significant *F*(1,96) = 8.477, *p* = 0.004, η^2^ = 0.081. As seen in Figure 4B, LTRY participants showed similar comfort levels when viewing original or 1/*f* amplitude images, collapsed across the phase condition, but HTRY participants had slightly lower levels of comfort when viewing the original amplitude images compared to the 1/*f* amplitude images, collapsed across the phase condition.

### Correlations Between TQ Scores and Comfort Level Across the Four Different Image Conditions

**FIGURE 5:**
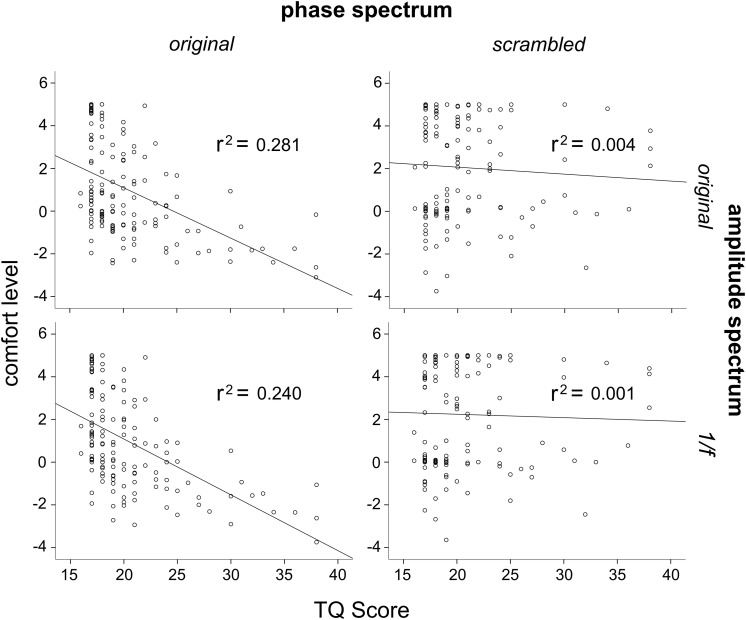
Correlations between scores on the TQ and average comfort levels for the four different image conditions. **Top left:** TQ scores correlated significantly with original trypophobic image comfort levels. **Bottom left:** TQ scores correlated significantly with original phase / 1/*f* amplitude image comfort levels. **Top right:** TQ scores were not correlated with scrambled phase / original amplitude image comfort levels. **Bottom right:** TQ scores were not correlated with scrambled phase / 1/*f* amplitude image comfort levels.

In order to further investigate the effects of phase and amplitude spectra on levels of comfort, correlation analysis was used to assess the relationship between participant’s TQ scores and their averaged level of comfort ratings for all image conditions. Three participants had outlier raw TQ scores (Z > 3.33), and were thus excluded from this analysis to be conservative (this did not affect results). Across the 144 participants, TQ scores were significantly negatively correlated with the original trypophobic image comfort levels, *r*(144) = −0.53, *p* < 0.001 (Figure 5, top left). This demonstrates validity of the TQ and replicates previous work (Le et al., 2015; Pipitone et al., 2017). TQ scores were also significantly negatively correlated with participants’ averaged comfort level for the original phase / 1/*f* amplitude images, *r*(144) = −0.485, *p* < 0.001 (Figure 5, bottom left). However, TQ scores were not significantly correlated with participant’s averaged comfort level for the scrambled phase / original amplitude images, *r*(144) = −0.064, *p* = 0.448 or the scrambled phase / 1/*f* amplitude images, *r*(144) = −0.031, *p* = 0.716 (Figure 5, top and bottom right, respectively), with these latter two results explaining almost no variance in comfort levels. Results from these analyses are summarized in Table 1.

**TABLE 1:** Correlations between averaged participant comfort levels given to the four image conditions and scores on the trypophobia questionnaire (TQ), scores from the bottom 33% (LTRY) and top 33% (HTRY) of the TQ.

	Comfort levels and TQ scores – Participant as level of analysis		
Image variable	TQ (*N* = 144)	LTRY (*N* = 49)	HTRY (*N* = 46)
Original phase/Original amplitude	−0.53**	0.044	−0.532**
Original phase/1/*f* amplitude	−0.485**	0.098	−0.488**
Scrambled phase/Original amplitude	−0.064	0.09	−0.116
Scrambled phase/1/*f* amplitude	−0.031	0.098	−0.061

**p < 0.001.

We also found no significant correlation between GAD-7 and TQ scores, *r*(144) = 0.012, *p* = 0.886. This replicates previous work showing that at among the general population, trypophobia does not seem to simply be a manifestation of generalized anxiety (Le et al., 2015; Pipitone et al., 2017; but see Vlok-Barnard and Stein (2017) for the comorbid relationship between trypophobia, depression and anxiety among trypophobic individuals).

To investigate how these results relate to participant’s TQ scores, we ran the same analyses described above on LTRY and HTRY participants. Among LTRY participants, TQ scores were not significantly correlated with original trypophobic image comfort levels, *r*(49) = 0.044, *p* = 0.766, comfort levels for the original phase / 1/*f* amplitude images, *r*(49) = 0.098, *p* = 0.505, comfort levels for the scrambled phase / original amplitude images, *r*(49) = 0.090, *p* = 0.540, or the scrambled phase / 1/*f* amplitude images, *r*(49) = 0.098, *p* = 0.504. Among HTRY participants, TQ scores were significantly correlated with original trypophobic image comfort levels, *r*(46) = −0.532, *p* < 0.001, and to the comfort levels for the original phase / 1/*f* amplitude images, *r*(46) = −0.488, *p* = 0.001. Critically, however, TQ scores and comfort levels were not significantly related to averaged comfort levels when viewing the scrambled phase / original amplitude images *r*(46) = −0.116, *p* = 0.444, or the scrambled phase / 1/*f* amplitude images, *r*(46) = −0.061, *p* = 0.685, with the latter results explaining almost no variance in comfort levels. These correlation analyses are summarized in Table 1.

## Discussion

Most work investigating trypophobia discusses the negative reactions when viewing these images as stemming from excess energy at mid-range spatial frequencies (Cole and Wilkins, 2013; Sasaki et al., 2017) which supports other work showing that deviations from natural 1/*f* amplitude spectra affects visual comfort (Fernandez and Wilkins, 2008; Juricevic et al., 2010; O’Hare and Hibbard, 2011; Hibbard and O’Hare, 2015). While these studies measure (Cole and Wilkins, 2013) or filter (Le et al., 2015; Sasaki et al., 2017) the amplitude spectrum of trypophobic images, the phase component was not manipulated or controlled systemically. The present study is the first to use Fourier analysis on trypophobic images to independently manipulate the phase and amplitude spectra in order to understand their relative contributions to levels of comfort. Our analyses clearly demonstrate that the phase spectrum plays a larger role in affecting viewing comfort than the amplitude spectrum (Figure 3). The main effect of phase (whether there were clustered images present or not) explained 24.9% of the variance in participant’s level of comfort, while the main effect of amplitude (excess energy at mid-range spatial frequencies) played a smaller role, explaining 9% of the variance in comfort. The interaction of phase and amplitude on levels of comfort was also significant, but only explained 6.1% of the variance in comfort levels. In other words, although the excess mid-spatial frequency energy in trypophobic images plays some role, the phase-dependent aspects (i.e., clusters of small objects, typically holes) played a much larger role.

By running the control experiment with non-trypophobic control images used in previous work (Cole and Wilkins, 2013) we ruled out the possibility that our main effect of phase scrambling (Figure 3) was a consequence of phase-scrambled images generally being more comfortable to view than natural images. In fact, we found that phase-scrambled control images were actually less comfortable to view on average than the original images. Therefore, we can interpret our phase-scrambling result (Figure 3) as consequence of removing phase-dependent image features like bumps or holes, which many participants find uncomfortable to view.

In exploring how levels of trypophobia would affect results, we observed that participants who scored in the top third of the TQ (HTRY) rated the original phase spectra images much lower in comfort than the phase scrambled images, but comfort ratings were comparable for both original and phase scrambled images among participants in the bottom third of the TQ (LTRY) (Figure 4A). As seen in Figure 4B, HTRY participants rated the original amplitude images lower in comfort compared to 1/*f* images, while LTRY participants rated original and 1/*f* images similarly. The TQ by phase interaction explained 36% of the variance in comfort ratings, while the TQ by amplitude interaction only explained 8.1% of the variance, again showing the larger impact of the phase spectra on comfort ratings, particularly for individuals who score high on the TQ.

In addition, scores on the full range of the TQ were used to investigate its relationship with comfort when viewing the four different image manipulation conditions. Our results demonstrated a strong negative correlation between comfort levels and TQ scores when viewing the original phase and amplitude images and when viewing the original phase / 1/*f* amplitude images (Figure 5, left two panels), both explaining 28.1 and 24% of the variance in comfort levels, respectively. However, there was no correlation between comfort levels and TQ scores when participants viewed the phase-scrambled / original amplitude images or phase-scrambled / 1/*f* amplitude images (Figure 5, right two panels), both explaining 0.4 and 0.1% of variance in comfort levels, respectively. What is more, among LTRY individuals, there was no relationship between TQ scores and comfort levels in any image condition, with all relationships explaining less than 1% of the variance in comfort levels. Even among HTRY individuals, comfort level variance was only explained by TQ scores when these participants viewed images with the phase structure present; TQ scores did not explain any meaningful variance in comfort levels (less than 3%) when participants viewed images comprising the original amplitude but scrambled phase structure (see Table 1). In summary, these findings suggest that even among trypophobic individuals, those who score higher on the TQ did not find images with the original amplitude spectrum intact more uncomfortable to view.

Two current evolutionary frameworks exist to explain the manifestation of trypophobia. One view is that trypophobic images mimic the patterns found on dangerous animals, such as snakes and spiders. Thus, having a negative reaction to those specific patterns may provide a survival advantage (Cole and Wilkins, 2013). This view posits that trypophobia is a misfiring of an otherwise adaptive *fear* response. The other viewpoint considers trypophobia as an adverse negative reaction to clustered patterns on the surface of the skin stemming from parasites and infectious diseases. Thus, trypophobia may be an exaggerated and overgeneralized, albeit adaptive response to avoid contact with diseased individuals (Kupfer and Le, 2017) with this viewpoint positing that trypophobia encompasses more of a *disgust* response. Recent work seems to support the latter infectious disease/disgust response. For example, Imaizumi et al. (2016) show a positive relationship between TQ scores and core disgust sensitivity. Kupfer and Le (2017) found that trypophobic individuals reported more disease-relevant pathogen avoidance remarks in open-ended questions when viewing trypophobic images. Yamada and Sasaki (2017) also showed that individuals with previous skin-related medical problems reported higher discomfort ratings toward trypophobic images compared to those who have no history of skin disease. Two other recent studies found greater unpleasantness ratings for faces of humans and animals when a trypophobic image (lotus seedpod) was superimposed on them compared to the trypophobic image viewed in isolation or when viewing the trypophobic image on inverted faces (Furuno et al., 2017, 2018).

Whether the discomfort elicited by trypophobic imagery is caused by fear or disgust, it is clear from our results that it is not strongly dependent on excess mid-range SF energy. In fact, one serious problem with the spectral energy hypothesis of trypophobia (Cole and Wilkins, 2013) is that as one varies viewing distance or image size, image spatial frequency content changes. For instance, if one doubles viewing distance, or halves image size, the spatial frequencies in the image approximately double. Therefore, visual discomfort elicited by potentially harmful stimuli (e.g., disease, or predators) would not be robust to changes in viewing distance or image size, making it a poor protective mechanism from an evolutionary perspective. In contrast, if the discomfort is cause by the patterns of holes or bumps rather than the SF content (as our results suggest), this would provide a more robust protective mechanism.

Some work has manipulated the phase and amplitude spectra of trypophobic images. Le et al. (2015) removed excess energy from trypophobic images (giving them 1*/f* natural image spectra but kept the original phase spectra intact) and showed that trypophobic comfort was unaffected compared to the original images (even among trypophobic individuals). Shirai and Ogawa (2019) found that trypophobic images enter visual awareness sooner than fear-related and neutral images, but scrambled phase spectra of the same trypophobic images (leaving the original amplitude spectrum intact) mitigated the “pre-perceptual” processing effect. However, during “post-perceptual” processing, phase-scrambled images were rated more negatively than other image types. This latter result, along with the current findings seem to suggest that although excessive mid-range spatial frequency energy may invoke slightly more unpleasantness than would otherwise be the case (see Figure 3), the excess energy has its largest effect on comfort through its interaction with the phase spectrum by enhancing the visibility of the clusters (compare Figure 1A upper and lower left panels). In other words, the amplitude spectrum, in and of itself, does not invoke a strong trypophobic response (compared to what is seen when the natural phase spectrum is present).

Despite the modest contribution of the amplitude spectrum to comfort levels when viewing trypophobic images, more generally images having spectral characteristics deviating from the 1/*f* spectra typical of most natural images have been shown to induce some visual discomfort (Wilkins et al., 1984; Fernandez and Wilkins, 2008; Juricevic et al., 2010; Penacchio and Wilkins, 2015). One hypothesis proposed to explain these findings is that images which induce visual discomfort elicit over-activity or *hyper-metabolism* in the visual cortex (Penacchio and Wilkins, 2015). This idea is supported by the studies like Wilkins et al. (1984) which demonstrated that the most discomforting gratings have mid-range spatial frequencies (∼3 cpd ±2 octaves) that are strongly represented in the primate visual cortex (De Valois et al., 1982) and to which the human visual system is most sensitive (Campbell and Robson, 1968). More recent work has demonstrated that a biologically plausible model of early visual cortex requires a larger number of active neurons to encode uncomfortable images than to encode comfortable images, also consistent with the hyper-metabolism hypothesis (Hibbard and O’Hare, 2015). As such, Le et al. (2020) recently showed that trypophobic images invoke larger hemodynamic responses in rear cortical areas among those who score high on the TQ. Inspired by our current findings with trypophobic imagery, it would be of great interest for researchers more broadly to consider possible interactions of amplitude and phase spectra in determining visual discomfort.

Although our study demonstrates a much stronger role for the phase spectrum than amplitude spectrum in determining visual discomfort in trypophobia, it does not tell us whether this is because the phase spectrum defines the semantic content (clusters of holes), or whether the phase distributions that define trypophobic images are a subset of a larger class of phase distributions which elicit visual discomfort. Although there is substantial literature on the effects of amplitude spectra (Juricevic et al., 2010) and orientation spectra (Ogawa and Motoyoshi, 2020) on visual comfort, to our knowledge no studies have systematically investigated the effects of phase spectra in isolation while controlling for other image properties. Our choice of a random, uniform phase spectrum for the control stimuli was meant to eliminate all forms of structure in the image, and this choice of phase spectrum has been used in a number of previous studies (e.g., Wichmann et al., 2006; Furuno et al., 2018; Shirai and Ogawa, 2019). However, this is by no means the only possible way to manipulate phase spectra, and more general investigations of the effects of phase structure on visual discomfort are of interest for future work.

For the present study, given the ecological relevance of disease avoidance, we hypothesize that it is the semantic content of trypophobic images which is primarily responsible for visual discomfort. This hypothesis is supported by recent work showing that super-imposing trypophobic stimuli onto pictures of upright faces induces more discomfort than viewing trypophobic stimuli in isolation or in other contexts (Furuno et al., 2017, 2018). In general, it is of great interest for future work to better understand the relative contributions of low-level image statistics and high-level semantic content in determining viewing comfort.

Finally, the contextual features of trypophobic images need to be further investigated in order to gain a better understanding for their specific impact on levels of comfort. For example, Le et al. (2015) showed no differences in comfort levels when viewing convex or concave images (i.e., bumps vs. holes) and suggest the “necessary but not sufficient” condition for unnatural image statistics to be the leading cause of trypophobia discomfort. Unfortunately, their image manipulations did not tease apart the phase and amplitude spectrum of the images, thus the amplitude properties were bound to the phase components. But, those images did have an underlying commonality; all had clusters comprised of circular objects. Would there be differences in comfort levels if the clusters were of non-circular objects? This phase specific aspect of trypophobic imagery might lend further credence to the parasitism model of infectious diseases, as many dangerous diseases (e.g., smallpox, typhus) involve clusters of roughly circular rashes or scabs on the skin (Kupfer and Le, 2017). What is more, the human visual system is sensitive to repetitive visual patterns (Conlon et al., 1999) thus levels of discomfort stemming from trypophobic images might be a consequence of repetitive clusters of roughly circular objects, which invokes an adaptive disgust response.

## Conclusion

While the current literature indicates excess energy at mid-range spatial frequencies as the defining feature of trypophobic images, our results suggest otherwise. The phase spectrum (independent of the amplitude spectrum) played a much larger role in determining comfort levels, compared to the amplitude spectrum (independent of the phase spectrum). While levels of trypophobia affected comfort levels attributed to images with the original phase spectrum intact, it did not play as big of a role in changing comfort levels as a function of image amplitude spectrum. Thus, the phase spectrum (sinusoidal waveform patterns) that comprise the image context seem to play a bigger role in the induction of trypophobic discomfort compared to the amplitude spectrum.

## Data Availability Statement

All data will be provided at the request of the reader by contacting the corresponding author.

## Ethics Statement

The studies involving human participants were reviewed and approved by the Florida Gulf Coast University Institutional Review Board. The patients/participants provided their written informed consent to participate in this study.

## Author Contributions

RP and CD conceived and designed the experiments and wrote the manuscript. CD manipulated the visual stimuli. RP collected and analyzed the data. Both authors contributed to the article and approved the submitted version.

## Conflict of Interest

The authors declare that the research was conducted in the absence of any commercial or financial relationships that could be construed as a potential conflict of interest.

## Footnotes

1 https://www.sona-systems.com/

2 https://www.checkbox.com/
